# Supplementary material for: “Resistance Is Futile”: A Pilot Study into Pseudoresistance in Canine Epilepsy
Source: Animals (Basel). 2023 Oct 6;13(19):3125. doi: 10.3390/ani13193125 (PMC10571656; doi:10.3390/ani13193125)
Supplement: Supplementary file 1 [file animals-13-03125-s001.zip › animals-2593695-supplementary.pdf]

Supplementary Materials:

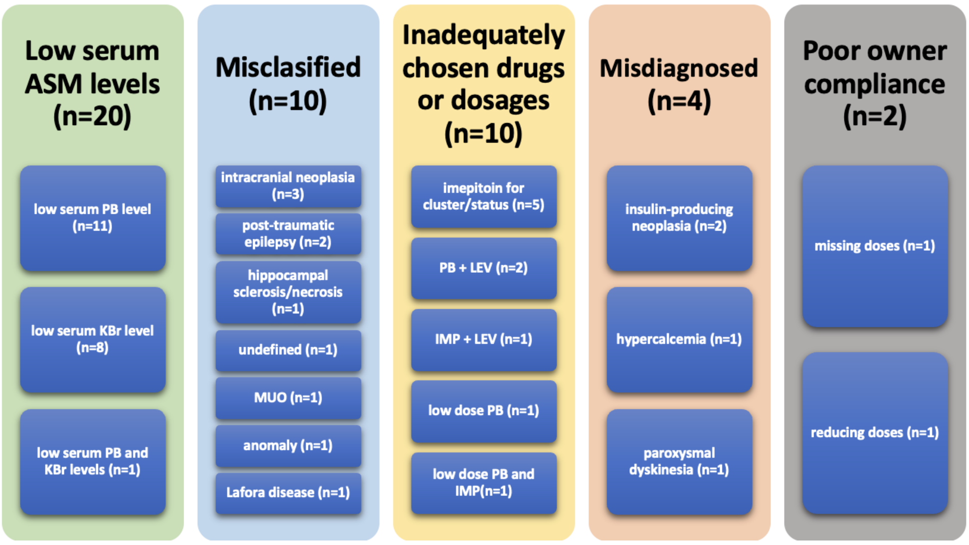

Figure S1. Distribution of patients within subcategories of different main pseudoresistance categories. (the figure does not show combinations of groups). Abbreviations: ASM = anti-seizure medication. PB = phenobarbital. KBr = potassium bromide. LEV = levetiracetam. IMP = imepitoin. MUO = meningoencephalitis of unknown origin.

Table S1. Response to changes in therapeutic protocols in different categories of patients with pseudoresistance. Abbreviations: ASM = anti-seizure medication.

CATEGORIES	RESPONSE	
Low ASM	Yes	n=15
	No	n=0
Misclassified	Yes	n=5
	No	n=1
Inadequately chosen drugs or dosages	Yes	n=6
	No	n=0
Misdiagnosed	Yes	n=4
	No	n=0
Poor owner compliance	Yes	n=2
	No	n=0
